# Effects of Seed Size and Cache Density on the Seed Fate of *Quercus wutaishanica* Mediated by Rodents

**DOI:** 10.3390/life14030286

**Published:** 2024-02-21

**Authors:** Jiming Cheng, Min Zhang, Xingfu Yan

**Affiliations:** 1 College of Biological Science and Engineering, North Minzu University, Yinchuan 750021, China; chengjiming@mails.ccnu.edu.cn; 2 School of Life Sciences, Central China Normal University, Wuhan 430079, China; minzhang@mails.ccnu.edu.cn; 3 Key Laboratory of Ecological Protection of Agro-Pastoral Ecotones in the Yellow River Basin, National Ethnic Affairs Commission of the People’s Republic of China, Yinchuan 750021, China

**Keywords:** oak, pilferage, re-hoarding, scatter-hoarding, seed density, seed mass

## Abstract

Animal-mediated seed dispersal is very important for plant population regeneration and the stability of forest ecosystems. Seed size and cache density are important factors for seed dispersal, but we still know little about seed size selection at different cache densities. Here, we conducted field experiments in a *Larix principis-rupprechtii* plantation in the Liupan Mountains in Ningxia province to investigate the effects of tag-marked *Quercus wutaishanica* seeds of different sizes and cache densities on predation and the scatter-hoarding behavior of rodents. The results showed lower proportions of intact in situ (IIS) and eaten in situ (EIS) large seeds than small seeds at all levels of cache density, with the exception of IIS seeds at a 6.25 seed·m^−2^ cache density. A higher proportion of small seeds were eaten after removal (EAR), but a higher proportion of large seeds were scatter-hoarded (SH) by rodents at most cache densities. Furthermore, rodents preferentially removed large seeds farther away for eating or scatter-hoarding. The IIS and EIS proportions of both large and small seeds declined, but the proportion of the two types of seeds that were EAR fluctuated, increasing with increasing cache density. Rodents preferred to increase the proportion of scatter-hoarding of large seeds with increasing cache density, whereas the proportion of scatter-hoarding of small seeds was maximized at a cache density of 6.25 seed·m^−2^. Both the eaten distance after removal (EDAR) and scatter-hoarded distance (SHD) increased with increasing cache density. These results suggest that large seeds are more likely to be scatter-hoarded and removed to longer distances than small ones. Rodents tended to reduce the seed proportion of EIS seeds and increased the proportion of seeds EAR and SH, and accordingly increased EDAR and SHD with increasing cache density. This study provides some scientific basis for animal-mediated seed dispersal.

## 1. Introduction

Rodents usually cache large amounts of food during times of adequate food supply to ensure a supply of nutrients to survive and reproduce during food shortages [1]. To reduce the risk of food pilferage, hoarders usually disperse food cache points over a large spatial range, i.e., by reducing the density of cache points [2,3]. The successful conservation of cached food is essential for hoarding animals to survive and reproduce during future food shortages. Characteristics such as plant seed size are important factors affecting animal dispersal and hoarding behavior. Seed size is an important indicator of seed quality and nutritional value [4]. Scatter-hoarding animals usually prefer to disperse and cache large seeds with high nutritional value, and carry large seeds to a farther distance, but consume small seeds with relatively low nutritional value in situ [2,5,6]. Because large seeds contain more nutrients, it helps hoarding animals to retrieve the same number of seed cache points during food shortages to obtain higher nutritional returns [7,8]. Therefore, there may be a trade-off mechanism between the benefits and inputs of hoarding different sizes of seeds. To maximize the benefits of hoarding behavior, animals must choose to preferentially disperse and scatter-hoard large seeds with more nutrients [9].

Olfaction and spatial memory are important ways for scatter-hoarding animals to retrieve cached food; olfaction plays an especially important role in searching for food cache points [10,11,12]. For example, Zhang and Zhang [10] found that the proportion of walnut (*Juglans mandshurica*) seeds that weakened olfactory information after embedding treatment was successfully searched by rodents. High-density food cache points are more conducive to successful animal search by random olfactory mining, that is, the pilferage rate of food cache points shows strong density dependence [13,14]. The maximum return per unit of time may also stimulate potential enthusiasm for consumers to search food cache points and pilfer seed; thus, seeds are more likely to survive at low densities at food cache points [4,15].

Liaodong oak (*Quercus wutaishanica*) is one of the important dominant tree species in warm temperate deciduous forests in China [16]. Regeneration based on seed dispersal, germination, and seedling establishment plays an important role in maintaining genetic diversity, population regeneration, and community stability [16]. Previous studies have found that Liaodong oak large seeds are more likely to be eaten after removal and scatter-hoarded, and that their dispersal distance is farther [17]. Yan et al. [18] indicated the cotyledon retention rate of high-density Liaodong oak annual seedlings was significantly lower than that of low-density seedlings under rodent predation pressure, that is, the strong olfactory information of dense seedling cotyledons can affect the search and hoarding behavior of rodents. This has an impact on seed and seedling fate and population regeneration. However, the interaction effect of seed size and cache point densities of Liaodong oak on rodent eating and scatter-hoarding behavior is still unknown. Therefore, in this study, we set different-size seeds (large and small seeds) and six cache point densities in Ningxia North China larch (*Larix principis-rupprechtii*) man-made forest, and study the influence of seed size and cache point density on rodent eating and scatter-hoarding behavior. The results could help to understand the mechanism of rodent-mediated plant seed dispersal, and reveal the cooperative evolutionary relationship between plant seeds and dispersal animals.

## 2. Material and Methods

### 2.1. Study Sites

The study site is located in the Datou forest area of Longtan Forest Farm of Liupanshan National Nature Reserve in Ningxia (35°23′54″ N, 106°21′12″ E, 1900 m above sea level) in a North China larch plantation planted for more than 35 years [16]. The study site is located in a temperate subhumid area with a continental monsoon climate. Annual precipitation is 676 mm, 60% of which is concentrated from July to September, and the annual evaporation volume is 1426 mm. The average annual temperature is 5.8 °C; the average temperature of the hottest month (July) is 17.4 °C, and that of the coldest month (January) is 7.0 °C. The soil is mainly gray-brown soil. The slope of the study site is about 35°, and the community canopy cover is above 85%. The community species composition, besides adult plants of North China larch, comprises young trees of *Q. wutaishanica*, *Populus davidiana*, *Betula platyphylla*, etc., and shrubs including *Euonymus phellomanus*, *Cotoneaster multiflorus*, *Fargesia nitida*, *Viburnum lobophyllum*, *Abelia dielsii*, and *Aralia chinensis*. The herbs mainly include *Gueldenstaedtia multiflora*, *Paeonia lactiflora*, *Poa annua*, and *Agropyron cristatum*. The rodents in this area mainly include *Niviventer confucianus*, *Apodemus agrarius*, *Microtus fortis*, and *Sciurotamias davidianus* [19].

### 2.2. Seed Collection and Marking

Liaodong oak seeds were collected from plants near the study site at the end of September. We collected and mixed seeds from 50 trees, then randomly divided them into 10 groups, and randomly selected 10 large and 10 small seeds in each group; a total of 100 large seeds and 100 small seeds were used for weighing. Seed size is represented by fresh seed mass [6]. The fresh masses of large and small seeds were 2.19 ± 0.47 g (mean ± standard deviation (Std), *n* = 100) and 0.80 ± 0.15 g (*n* = 100), respectively. A small hole was drilled at the base of each seed with an electric drill of 0.5 mm in diameter; then, a copper wire with a diameter of 7.5 cm wrapped with a plastic protective layer was put through the hole. Seeds were connected with a red plastic label of 2.5 cm by 1.2 cm (including copper wire mass 0.17 ± 0.002 g, *n* = 100), and the label recorded the information of the location, burial point, and location coordinates [20]. We put the marked seeds in a breathable nylon mesh bag and stored them in a 4 °C refrigerator. After the seeds were eaten by rodents, the labels were discarded at the consuming area, and the labels attached to the seeds that were cached (or discarded) after dispersal were usually exposed at the surface, and the fate types of the seeds could be distinguished by searching for the labels during the field survey.

### 2.3. Experimental Design

**Figure 1:**
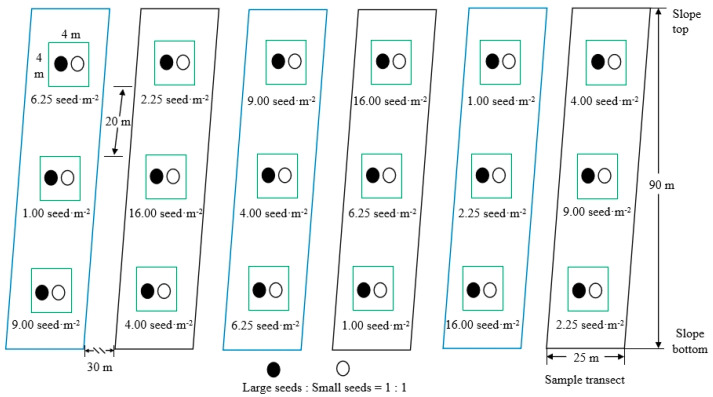
Distribution diagram of release plots in sample transect.

In the North China larch plantation, we set up 6 sample transects about 30 m apart to randomly arrange 6 seed cache density treatments (to exclude the interference of species and density differences of rodents at different transects). Three sample plots (4 m × 4 m) were set at about 20 m apart in each transect. Three replicates were set for each cache point of the same density. Within 3 random sample plots of 6 transects, the ratio of large seeds to small seeds was 1:1, and 16, 36, 36, 64, 100, 144, and 256 Liaodong oak seeds of different sizes were evenly buried; that is, the cache density of each plot of seeds was 1.00, 2.25, 4.00, 6.25, 9.00, and 16.00 seed·m^−2^, respectively (Figure 1). The total number of seeds in the test was as follows: 6 cache densities (16 + 36 + 64 + 100 + 144 + 256) × 3 repeats (sample plot) = 1848 seeds.

### 2.4. Field Investigation

Taking the center of the sample plot as the origin, each plot was equally divided into four quadrants, and the large and small seeds were evenly buried in the quadrant of the sample plot, and the specific plot and quadrant of each seed was recorded. When the seeds were buried, the litter and surface soil in the sample plot were cleaned up, the seeds were put into the soil layer, and then the seeds and labels were covered with about 2.5 cm of fine soil. After the seeds were buried, the excess soil and litter were restored to the state before seed burial. Each seed burial point was checked at the 2nd, 5th, 9th, 14th, 21st, 28th, and 60th days after seed burial treatment, and the number and label information were recorded for the retained seeds at the original burial point and dispersed seeds within a radius of 25 m around the center of each plot [3,21]. The label information of the collected seeds was recorded, and the distance between the seed label and the corresponding burial point was determined with a measuring tape. After each inspection, the seeds remaining at the initial cache point were restored to their original buried state.

### 2.5. Seed Fates

Based on the field survey data, and according to the definition of seed fates by Zhang [3], seed fates were divided into five types: (1) intact in situ (IIS): the seeds remain intact at the seed release point, representing seeds that are not eaten and dispersed by rodents; (2) eaten in situ (EIS): seeds are eaten at the release point by rodents, and seed labels or fragments are scattered at the seed release point after the seeds are eaten, meaning that the seeds are eaten by rodents before dispersal; (3) eaten after removal (EAR): the seeds are eaten after being removed from the release point, and seed labels or fragments are scattered at the consuming point, meaning that the seeds are eaten by rodents after dispersal; (4) scatter-hoarded (SH): the seeds are moved away from the release point and buried in soil, dead leaves, grass, or other matrixes, indicating that the seeds are dispersed by rodents; (5) missing (M): the seeds are not found after being removed from the release point, indicating that the seed fates cannot be determined. The number of large and small seeds with different fates at the densities of each cache point were counted, and the eaten distance after removal (EDAR) and scatter-hoarded distance (SHD) were calculated. Then, we calculated the intact in situ rate and the percentage of seeds with different fates of the total number of seeds.

### 2.6. Data Analysis

We computed generalized linear models (GLMs) for the effects of seed size and cache density on seed fates (percentage) and seed dispersal distance. We used seed size and cache density as explanatory variables, plot as a random effect, and seed fates and dispersal distance as dependent variables. We fitted generalized linear models for Gaussian variables (family = Gaussian; link = identity) and count variables (family = Poisson; link = log) in the “lmer4 package” [22]. Furthermore, we used generalized linear models to test the pairwise interaction between seed size and cache density on seed fates and dispersal distance. These analyses were performed with R 4.2.2 [23]. The least significant difference method (LSD) was used to evaluate the effect of the seed sizes and cache point densities on seed intact in situ rate, the percentage of seeds with different fates, the eaten distance after removal, and scatter-hoarded distance of seeds in SPSS (Version 21.0) [16]. All data are expressed as the mean ± standard deviation (Std). The normality test and the standardization of data were conducted in SPSS 21.0. We used SigmaPlot (Version 10.0) to make all figures.

## 3. Results

### 3.1. Seed Intact In Situ Rate and Dynamics

**Figure 2:**
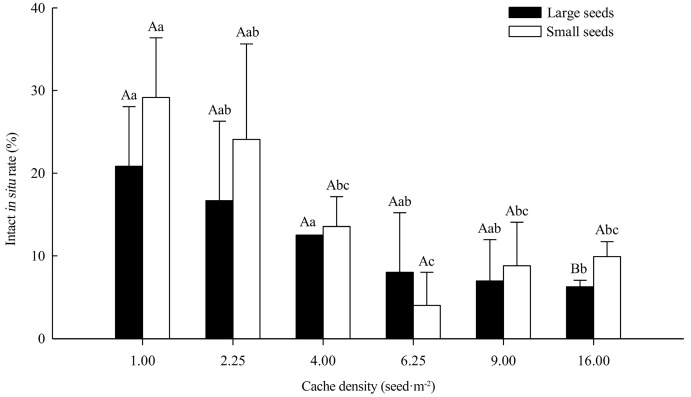
Intact in situ rate of different-sized *Quercus wutaishanica* seeds under different cache densities at experimental site of *Larix principis-rupprechtii* plantation in Liupan Mountains. Different capital letters and small letters indicate significant difference between different-sized seeds within the same cache density and among different cache densities within the same-size seeds at *p* < 0.05, respectively. The data are presented as mean ± Std. The same below.

Seed size had no significant effect on intact in situ rate, but large seeds were lower than small seeds in different cache densities except 6.25 seed·m^−2^, and intact in situ for different-size seeds only varied significantly at 16.00 seed·m^−2^ density (*p* < 0.05). Cache density had a significant effect on intact in situ rate (*p* < 0.01). However, the interaction between the seed size and cache density had no significant effect on the intact in situ rate. The intact in situ rate of large seeds gradually decreased with increasing cache density, and the intact in situ rates were higher at 1.00, 2.25, and 4.00 seed·m^−2^ densities (20.8%, 16.7%, and 12.5%, respectively); the 1.00 and 4.00 seed·m^−2^ densities were significantly greater than the 16.00 seed·m^−2^ density (6.3%) (*p* < 0.05). The intact in situ rate of small seeds at 1.00 seed·m^−2^ (29.2%) was significantly greater than other densities, except 2.25 seed·m^−2^ (*p* < 0.05), with a minimum at 6.25 seed·m^−2^ density (4.0%) (Figure 2).

### 3.2. Seed Fates

The eaten in situ rates of Liaodong oak seeds were significantly affected by seed size (*p* < 0.01). The eaten in situ rates of small seeds were higher than large seeds at all densities. And, the small seeds were approximately 14.0 and 9.7 times that of large seeds at 9.00 and 16.00 seed·m^−2^ densities, respectively (Table 1). Cache density had no significant effect on the eaten in situ rate of large seeds, and the overall trend was relatively high eaten in situ rates at low densities, and low eaten in situ rates at high densities. The eaten in situ rate of small seeds at 1.00 seed·m^−2^ density (25.00%) was significantly greater than 6.25, 9.00, and 16.00 seed·m^−2^ densities (*p* < 0.05).

The eaten after removal rate was significantly influenced by seed size and cache density (*p* < 0.01). The eaten after removal rates of large seeds were lower than small seeds in all densities except 1.00 seed·m^−2^ density, and large seed rates were significantly lower than small seeds at 16.00 seed·m^−2^ density (*p* < 0.01). The eaten after removal rate of large seeds increased first and then decreased later with increasing cache density. The eaten after removal rate of small seeds was at its minimum at 1.00 seed·m^−2^ density, and significantly lower than 4.00 and 16.00 densities (*p* < 0.05 and *p* < 0.01), and there were also significant levels at 4.00 and 9.00 densities (*p* < 0.05).

The scatter-hoarding rate was significantly influenced by seed size and cache density (*p* < 0.01). The scatter-hoarding rates of large seeds were higher than small seeds at all densities (except 6.25 seed·m^−2^), and large seed rates were significantly higher than small seed rates at 16.00 seed·m^−2^ density (*p* < 0.01). The scatter-hoarding rate of large seeds had the lowest density at 1.00 seed·m^−2^ density (4.2%), which increased with increasing cache density. Small seeds had the highest density at 6.25 seed·m^−2^ density (13.3%), which was significantly greater than 4.00 seed·m^−2^ density (*p* < 0.05).

The missing rate was significantly influenced by seed size and cache density (*p* < 0.01). The missing rate of large seeds was significantly higher than small seeds at 16.00 seed·m^−2^ density (*p* < 0.01), but was significantly lower than small seeds at 9.00 seed·m^−2^ density (*p* < 0.01). The missing rate of large seeds was not significant between different cache densities. The missing rate of small seeds at 9.00 seed·m^−2^ density was significantly higher than at 2.25, 4.00, and 16.00 seed·m^−2^ densities (*p* < 0.01, *p* < 0.05, and *p* < 0.05), and the 6.25 seed·m^−2^ density was also significantly higher than the 2.25 seed·m^−2^ density (*p* < 0.05).

### 3.3. Seed Dispersal Distance

Seed size and cache density had significant effects on the eaten distance after removal (seed size: *p* < 0.01; cache density: *p* < 0.01) and scatter-hoarding distance (seed size: *p* < 0.01; cache density: *p* < 0.01). The eaten distance after removal of large seeds was greater than small seeds, and especially significantly greater than small seeds at 4.00, 6.25, and 9.00 seed·m^−2^ densities (*p* < 0.05, *p* < 0.01, and *p* < 0.05) (Table 2). The eaten distance after removal of large and small seeds increased with increasing density. The eaten distance after removal of large seeds at 6.25 seed·m^−2^ density was significantly greater than 1.00, 2.25, 4.00, and 16.00 seed·m^−2^ densities (*p* < 0.05, *p* < 0.01, *p* < 0.05, and *p* < 0.05) (Table 2). The eaten distance after removal of small seeds at 6.25 seed·m^−2^ density was significantly greater than 1.00, 2.25, and 4.00 seed·m^−2^ densities (*p* < 0.01, *p* < 0.01, and *p* < 0.05). 

The scatter-hoarding distance of large seeds was significantly greater than small seeds at 2.25, 4.00, and 6.25 seed·m^−2^ (all *p* < 0.05). The scatter-hoarding distance of large and small seeds increased with increasing density. Specifically, the scatter-hoarding distance of large seeds at 6.25 seed·m^−2^ density (10.31 m) was significantly greater than 1.00, 2.25, and 4.00 seed·m^−2^ densities (all *p* < 0.05), and at 9.00 seed·m^−2^ density was significantly greater than 2.25 and 4.00 seed·m^−2^ densities (all *p* < 0.01). The scatter-hoarding distance of small seeds at 9.00 seed·m^−2^ density was significantly greater than 2.25 and 4.00 seed·m^−2^ densities (*p* < 0.05 and *p* < 0.01).

## 4. Discussion

Hoarding animals usually cache food in a large spatial range, i.e., reduce the cache density to reduce the risk of cached food being pilfered [24,25]. At the same time, animals enhance their memory of cache points through the repeated excavation of cache food and optimizing cache management conditions, thus giving them an advantage over pilferers when searching for cached food [10,24,26]. This behavior promotes the evolution of scatter-hoarding behavior in animals [12,24]. The large number of competitors will inevitably increase the risk of cached food being pilfered, and pilfering cached food from other individuals has become a potential foraging strategy in rodents [24]. Wang et al. [4] found that seed size was positively correlated with the possibility of repeated pilferage and caching because the potential motivation of rodents to search and pilfer food cache points increased with the nutritional value of seeds, but rodents eating and scatter-hoarding large seeds may increase investments in time and energy. Therefore, to retrieve cache seeds and search for less food cache points with the same nutritional value, animals usually choose to cache larger seeds, which can also effectively compensate for the loss of cached food that is pilfered [27,28].

This study found that the intact in situ rates of large seeds were lower than small seeds in other cache densities except 6.25 seed·m^−2^, and the eaten in situ rates of large seeds were lower than small seeds in all cache densities. The eaten after removal rates of large seeds were lower than small seeds at all cache densities except 1.00 seed·m^−2^, while the scatter-hoarding rates of large seeds were higher than small seeds at all cache densities except 6.25 seed·m^−2^. These results show that the nutritional value of seeds and the cost input of rodents for eating and dispersing seeds are important factors that affect rodent foraging strategies. Rodents consumed large seeds faster and prioritized eating small seeds in situ or after removal after pilfering cache points because the lower nutrient storage of small seeds may not be enough to compensate for the time and energy input of dispersal and hoarding [29]. Hoarding more large seeds could ensure the retrieval of cached seeds during food shortages, thus obtaining higher nutritional returns [7,8]. To maximize the benefits of consuming and scatter-hoarding food, rodents may have to trade-off between caching different sizes of seeds with the input required for foraging behavior processes [30]. The input and predation risks of consuming and dispersing large seeds may be increased, but rodents still prefer to consume and disperse large seeds, and the potential motivation to search for seed cache points will be enhanced with the increasing nutritional value of the seeds [4]. Although, in this study, the eaten distance after removal and scatter-hoarding distance of different sizes of seeds only differed significantly for some cache densities, the eaten distance after removal and scatter-hoarding distances of large seeds were greater than small seeds to different degrees. The results showed that, to reduce the risk of food being pilfered at newly established cache points, rodents choose to disperse large seeds with high nutritional value to farther cache points. For rodents, caching large seeds in a larger space may increase time and energy costs and increase the risk of predation, and the willingness to pay these costs may be related to the higher nutritional return of large seeds [31]. Moreover, the higher missing rate of large seeds at most cache points may also reflect the nutritional differences between large and small seeds, because large seeds may have been dispersed by rodents beyond our search range.

Scatter-hoarding rodents may search food cache points through olfaction and random digging, which are important means by which consumers search for food [11]. In this study, we simulated the way that rodents disperse and hoard seeds, and the seeds were also buried in the soil; that is, the visual signals of the food cache points were disguised and masked, and the pilferers could only search the seed cache points by olfaction. Stronger olfactory signals at high-density cache points facilitate the successful food search of rodents, and the pilferage rate of seed cache points shows density dependence [4,14,15]. And, pilferers may work harder to search for the next one after occasionally searching a food cache point [13,27]. In this study, the intact in situ rate of Liaodong oak seeds of different sizes decreased with increasing cache density, indicating that the nutritional return of foraging behavior per unit of time and energy input may be high, thus stimulating the potential enthusiasm of individuals to search for and pilfer food cache points [4]. On the contrary, consumers may give up the search due to low cache density, so the intact in situ rate of seeds and cache density have a negative correlation, i.e., negative density dependence [14,15]. This result is consistent with the study conducted by Zhang and Zhang [32] on sunflower (*Helianthus annuus*) seeds under fence conditions. Liu et al. [33] found that the size of seed cache points of Korean pine (*Pinus koraiensis*) significantly affected the proportion of seeds found by rodents (*Tamias sibiricus*), which may be related to the strong odor of large seed cache points. This is also similar to way in which high cache density provided stronger olfactory signals for rodents in this study.

In this study, we found that the eaten in situ rate of large and small Liaodong oak seeds showed a trend of decrease first, and then increase with the increase in cache density, and the eaten after removal rate showed an increasing trend (with the highest at 4.00 seed·m^−2^ density). The scatter-hoarding rate of large seeds gradually increased with cache density, and the scatter-hoarded rate of small seeds was relatively high at high cache density. These results show that rodents usually choose to eat seeds in situ at low cache density after pilfering food to satisfy their daily energy demand. With increasing cache density, to avoid missing the opportunity to harvest rich food resources, rodents may increase food caches under conditions of relatively adequate food availability [8]. Thus, the amount of food intake is increased during seed dispersal to compensate for the excessive energy consumption during massive food searches and caching. In addition, animals appropriately reducing the eaten in situ rate and increasing the eaten after removal rate can also reduce the risk of predation during the foraging process [19]. However, as the cache density of seed continues to increase, the ability of rodents to search and scatter-hoard seeds increases. This may increase the intact in situ rate of rodents to compensate for the demand of energy consumption and improve scatter-hoarding rate while appropriately reducing the eaten after removal rate. These results are consistent with the predictions of the predator dispersal hypothesis and the reciprocal pilferage hypothesis [8,26,34,35,36].

In this study, the eaten distance after removal and scatter-hoarding distance increased with cache density, but after cache densities over 6.25 (large seeds) and 9.00 seed·m^−2^ (small seeds), the eaten distance after removal and scatter-hoarding distance gradually decreased. This result may be related to the “saturation effect” on pilferers due to the relatively adequate food supply at high cache densities [37,38]. And, optimal cache distance determines that the pilferer may minimize the cost input of dispersal and cache food under relatively adequate food supply [13,37]. Moreover, the food pilfered by competitors can be compensated by pilfering cached food from other individuals. This study was conducted in the autumn when seed resources were relatively abundant. The superposition effects of high-density test seeds (cache points) and the input of relatively abundant food resources may reduce the value of cache seeds and thus weaken the long-distance seed dispersal of rodents. Moreover, during the field investigation period of the study there was a large number of deciduous trees and the litter cover affected the successful seed searches of rodents. Thus, the missing rate of the test seeds was high. The study results partly reflect the eating and scatter-hoarding behaviors of rodents at different cache densities.

Obviously, rodents tend to scatter-hoard more large seeds and disperse large seeds to longer distances because large seeds have more nutrients. This indicates that large seeds have a greater chance of germination and seedling establishment, which facilitates the regeneration of the plant population. The higher the seed density, the more attractive it is for rodents to disperse seeds, reducing the probability of seed death due to density restriction. However, higher density leads to the “predator saturation effect”, reducing the chance of seed germination. These results imply that large seeds and suitable seed density are more favorable for improving plant population regeneration and forest ecosystem stability. In forest management and conservation, we choose to plant excellent tree species as much as possible and maintain a certain planting density, which is not only beneficial for growing more large seeds, but also to ensure moderate seed density, which is beneficial for the regeneration of plant populations.

## 5. Conclusions

This study showed that seed size and cache density are important factors affecting the consumption and dispersal of Liaodong oak seeds by rodents. Large seeds were consumed by rodents faster than small seeds at all cache points. The eaten in situ and eaten after removal rates of large seeds were generally lower than small seeds, and the scatter-hoarding rate was higher than small seeds. Both the eaten distance after removal and scatter-hoarding distances of large seeds were greater than for small seeds. With the increase in density of cache points, the eaten in situ rate decreased and eaten after removal rate increased. The scatter-hoarding rate for high densities of cache points was relatively high. The eaten distance after removal and scatter-hoarding distance increased with the density of cache points. Higher-density seed cache points may have a “saturation effect” on animals and decrease the seed dispersal distance. In the future, it is essential to explore the influence of more factors on seed dispersal.

## Figures and Tables

**Table 1:** Fates of *Quercus wutaishanica* seeds with different sizes under various cache densities.

Density (Seed·m^−2^)	EIS		EAR		SH		M	
	Large Seed	Small Seed	Large Seed	Small Seed	Large Seed	Small Seed	Large Seed	Small Seed
1.00	4.17 ± 7.22 a	25.00 ± 12.50 a	16.67 ± 14.43 a	8.33 ± 14.43 c	4.17 ± 7.22 a	0.00	54.17 ± 7.22 Aa	37.50 ± 21.65 Aac
2.25	3.70 ± 3.21 Aa	12.96 ± 6.42 Aab	22.22 ± 14.70 Aa	48.15 ± 11.56 Aabc	11.11 ± 5.56 Aa	3.70 ± 3.21 Aab	46.30 ± 26.25 Aa	11.11 ± 0.00 Ac
4.00	2.08 ± 3.61 Aa	5.21 ± 3.61 Ab	29.17 ± 25.26 Aa	60.42 ± 6.51 Aa	11.46 ± 7.86 Aa	1.04 ± 1.80 Ab	44.79 ± 20.81 Aa	19.79 ± 7.86 Abc
6.25	0.67 ± 1.15 Aa	4.67 ± 1.15 Ab	22.00 ± 4.00 Aa	42.67 ± 19.63 Aabc	13.33 ± 4.16 Aa	13.33 ± 4.16 Aa	56.00 ± 12.49 Aa	35.33 ± 12.70 Aab
9.00	0.46 ± 0.80 Aa	6.48 ± 2.12 Ab	12.95 ± 9.76 Aa	38.43 ± 10.42 Abc	14.81 ± 11.31 Aa	8.80 ± 5.78 Aab	6.48 ± 2.12 Ba	37.50 ± 2.78 Aa
16.00	0.78 ± 0.35 Aa	7.55 ± 1.63 Ab	18.75 ± 7.69 Ba	53.65 ± 2.26 Aab	15.36 ± 5.86 Aa	8.85 ± 5.86 Bab	58.85 ± 11.38 Aa	20.05 ± 1.90 Bbc

Different capital letters and small letters indicate significant difference between different-sized seeds within the same cache densities and among different densities within same-size seeds at *p* < 0.05, respectively. The data are presented as mean ± Std. The same below.

**Table 2:** Eaten distance after removal and scatter-hoarding distance of different-size *Quercus wutaishanica* seeds under different cache densities (seed·m^−2^).

Cache Density (Seed·m^−2^)	SEED Size	EDAR	SHD
		Mean ± Std (Number)	Mean ± Std (Number)
1.00	Large seed	2.03 ± 1.96 c (*n* = 4)	1.10 ± 0 b (*n* = 1)
	Small seed	0.15 ± 0.09 b (*n* = 2)	—
2.25	Large seed	2.00 ± 1.55 Ac(*n* = 14)	4.54 ± 1.56 Ab (*n* = 6)
	Small seed	0.88 ± 0.49 Ab (*n* = 27)	0.65 ± 0.38 Bb (*n* = 2)
4.00	Large seed	5.43 ± 1.25 Abc (*n* = 29)	5.75 ± 1.78 Ab (*n* = 11)
	Small seed	1.39 ± 0.71 Bb (*n* = 59)	0.40 ± 0 Bb (*n* = 1)
6.25	Large seed	9.76 ± 1.70 Aa (*n* = 36)	10.31 ± 3.39 Aa (*n* = 20)
	Small seed	4.39 ± 0.18 Ba (*n* = 66)	4.19 ± 0.88 Ba (*n* = 20)
9.00	Large seed	7.58 ± 1.49 Aabc (*n* = 35)	7.35 ± 4.21 Aa (*n* = 32)
	Small seed	2.49 ± 1.75 Bab (*n* = 91)	5.55 ± 0.68 Aa (*n* = 19)
16.00	Large seed	5.87 ± 1.19 Abc (*n* = 77)	6.63 ± 1.00 Aab (*n* = 60)
	Small seed	2.07 ± 0.40 Aab (*n* = 213)	2.55 ± 0.82 Aab (*n* = 34)

Different capital letters and small letters indicate significant difference between different-sized seeds within the same cache densities and among different densities within same-size seeds at *p* < 0.05, respectively.

## Data Availability

Dataset available on request from the authors.
